# Efficient graph convolutional networks for seizure prediction using scalp EEG

**DOI:** 10.3389/fnins.2022.967116

**Published:** 2022-08-01

**Authors:** Manhua Jia, Wenjian Liu, Junwei Duan, Long Chen, C. L. Philip Chen, Qun Wang, Zhiguo Zhou

**Affiliations:** ^1^School of Integrated Circuits and Electronics, Beijing Institute of Technology, Beijing, China; ^2^Department of Neurology, Xuanwu Hospital, Capital Medical University, Beijing, China; ^3^College of Information Science and Technology, Jinan University, Guangzhou, China; ^4^Faculty of Science and Technology, University of Macau, Taipa, Macau SAR, China; ^5^School of Computer Science and Engineering, South China University of Technology, Guangzhou, China

**Keywords:** seizure prediction, EEG, GCN, geometric deep learning, wearable devices

## Abstract

Epilepsy is a chronic brain disease that causes persistent and severe damage to the physical and mental health of patients. Daily effective prediction of epileptic seizures is crucial for epilepsy patients especially those with refractory epilepsy. At present, a large number of deep learning algorithms such as Convolutional Neural Networks and Recurrent Neural Networks have been used to predict epileptic seizures and have obtained better performance than traditional machine learning methods. However, these methods usually transform the Electroencephalogram (EEG) signal into a Euclidean grid structure. The conversion suffers from loss of adjacent spatial information, which results in deep learning models requiring more storage and computational consumption in the process of information fusion after information extraction. This study proposes a general Graph Convolutional Networks (GCN) model architecture for predicting seizures to solve the problem of oversized seizure prediction models based on exploring the graph structure of EEG signals. As a graph classification task, the network architecture includes graph convolution layers that extract node features with one-hop neighbors, pooling layers that summarize abstract node features; and fully connected layers that implement classification, resulting in superior prediction performance and smaller network size. The experiment shows that the model has an average sensitivity of 96.51%, an average AUC of 0.92, and a model size of 15.5 k on 18 patients in the CHB-MIT scalp EEG dataset. Compared with traditional deep learning methods, which require a large number of parameters and computational effort and are demanding in terms of storage space and energy consumption, this method is more suitable for implementation on compact, low-power wearable devices as a standard process for building a generic low-consumption graph network model on similar biomedical signals. Furthermore, the edge features of graphs can be used to make a preliminary determination of locations and types of discharge, making it more clinically interpretable.

## 1. Introduction

Epilepsy is a brain disorder caused by abnormal brain activity. There are approximately 50 million people with epilepsy of all ages around the world (Organization, 2019). It is worth noting that these patients are at three times the risk of premature death than the general population, with sudden death in epilepsy, status epilepticus, unintentional injuries, and suicide being the most common causes. The leading and potentially preventable cause of death in people with epilepsy. In addition, people with epilepsy often have cognitive, psychological, and interpersonal impairments. Electroencephalogram (EEG) is widely used in healthcare (Duan et al., 2012) and epilepsy-related departments, especially in primary hospitals, and has become the key research data for epilepsy diagnosis and treatment due to its low cost, ease of use, tolerable movement restrictions, painless and comfortable procedure, and long-term monitoring. Although many advances have been made in epilepsy detection, there are few studies related to epilepsy prediction due to its complexity and difficulty. However, effective daily prediction of seizures is an important support for timely administration of fast-acting drugs, avoiding unintended harm, and relieving tension.

Developments in the field of seizure prediction have been greatly driven by the establishment of seizure EEG databases, the holding of international epilepsy prediction competitions, and the standardization of performance evaluation of algorithms (Kuhlmann et al., 2018). In terms of algorithms, deep learning has become the most popular research method for seizure prediction. However, traditional deep learning models are often used, such as Convolutional Neural Networks (CNN) (Rosas-Romero et al., 2019; Sharan and Berkovsky, 2020; Wang et al., 2020; Li et al., 2021d; Ozdemir et al., 2021; Usman et al., 2021), Recurrent Neural Networks (RNN) (Tsiouris et al., 2018; Li et al., 2021d; Usman et al., 2021), and new deep learning models, such as multi-view CNN (Liu et al., 2019), multi-time scale CNN (Qi et al., 2021), semi-expanded CNN (Hussein et al., 2021), and Transformer (Hussein et al., 2022). Can only process Euclidean grid data, often EEG data or the feature is represented as a real number matrix. However, this representation does not take into account the structural connectivity characteristics of EEG data, so a large number of model parameters requires more storage and computational consumption when automatically extracting features. Concerning this issue, some researchers use pruning (Zhao et al., 2021a), Neural Architecture Search (NAS) (Lee et al., 2020; Dong et al., 2021; Li et al., 2021a; Xue et al., 2021; Yang et al., 2021c; Zhao et al., 2021a; Wang et al., 2022), and other methods to simplify CNNs. Although a simplified model can eventually be obtained, it is still challenging to ensure the performance of the prediction.

**Figure 1:**
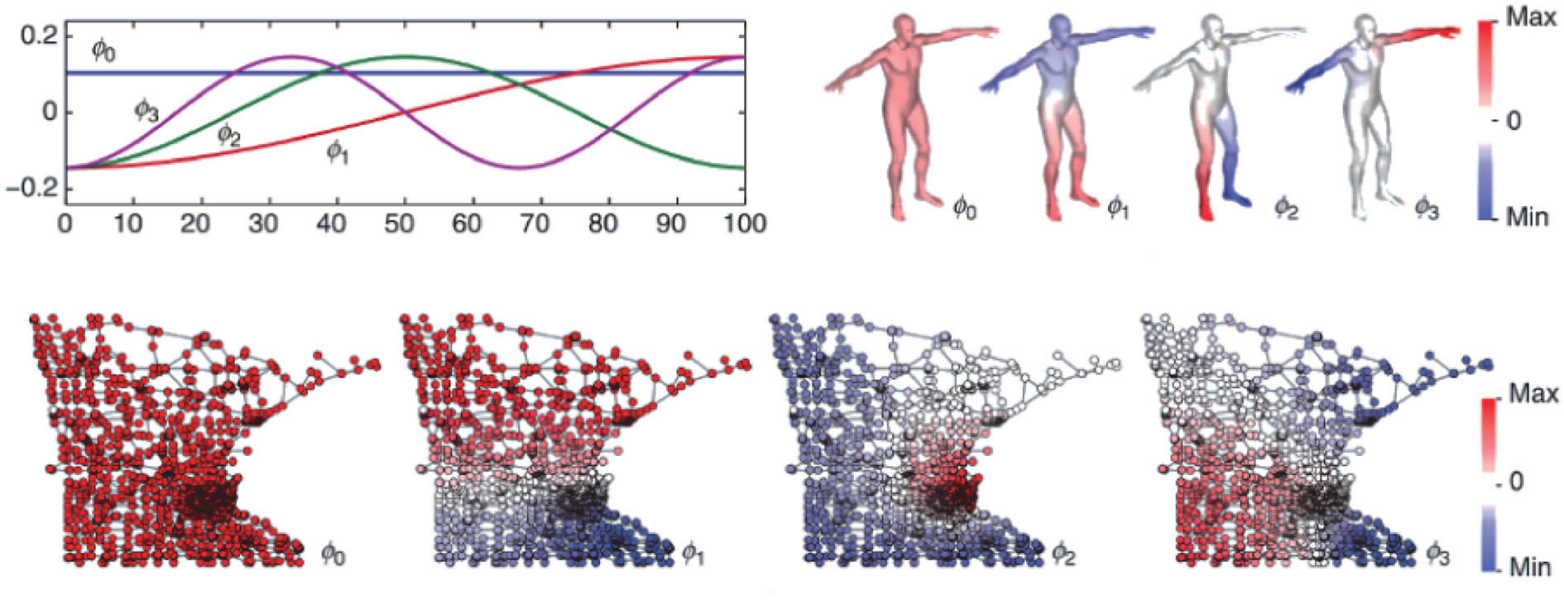
Example of the four Laplacian eigenfunctions ϕ_0_, ..., ϕ_3_ on a Euclidean domain (1D line, top left) and non-Euclidean domains (human shape modeled as a 2D manifold, top right; and Minnesota road graph, bottom). In the Euclidean case, the result is the standard Fourier basis comprising sinusoids of increasing frequency. The citation requested by third-party rights holders is as follows: ©[2017] IEEE.

Graphs are a way of representing entity relationships and structured data that are ubiquitous in the real world, such as social networks, business networks, biological networks, transportation networks, and knowledge graphs. A graph consists of some nodes and edges connecting these nodes and contains rich potential value by its complex structure. Human brains are massively complex networks of functional and structural domains, and graph theory provides a new perspective on their analysis. Figure 1 (Bronstein et al., 2017) visually shows the difference between Euclidean and non-Euclidean domains (manifolds and graphs). Specifically, the flatness of Euclidean spaces means that certain operations require many dimensions and complex computations to perform, whereas non-Euclidean spaces may perform these operations more flexibly with fewer dimensions. The graph structure breaks the uniform distribution of the commonly used Euclidean grid structure and can better represent the structural connections and functional realization of brains (Wein et al., 2021). It has been used in the diagnosis of autism spectrum disorders (Yang et al., 2021a), fMRI analysis (Li et al., 2019b, 2021b), brain network analysis (Wu et al., 2021; Royer et al., 2022), brain-computer interface decoding (Feng et al., 2021; Che et al., 2022), emotion classification (Liu et al., 2022), and epilepsy detection (Zhao et al., 2021b). Studies of graph networks for seizure prediction are rare and often focus on complex variants of graph networks while ignoring the model's scale (Lian et al., 2020) or building models that are too large (Dissanayake et al., 2021b; Li et al., 2021c). Lian et al. (2020) proposed a global-local graph convolutional network (Kipf and Welling, 2016) for robust seizure prediction. Compared with the sEEG signal, the processed iEEG signal has more channels, higher sampling frequency, and less artifact interference. While providing richer and cleaner information for seizure prediction, the premise of surgically implanting a signal collector also limits the application scope of the algorithm and ignores the need for a reduced model size in practical application scenarios. Furthermore, they believed that it is almost impossible to build a proper general prior map to predict seizures, so they proposed to use deep learning to build the prior graph. Li et al. (2021c) proposed a spatiotemporal spectral hierarchical graph convolutional network with semi-supervised active learning for seizure prediction. They used sEEG and also fully utilized deep learning to synthesize prior graphs, for which there was a problem of building a model that was too large. A GCN seizure predictor using sEEG was also proposed by Dissanayake et al. (2021b). They use deep learning to further synthesize the prior graph based on the physical connection derived graph, and the problem of the large model size still exists. However, mobile portable seizure predictors loaded with reduced-scale models are particularly important for patients with refractory epilepsy who are inoperable or ineffective for surgical resection.

In this article, each sample is modeled as a graph, with nodes representing channels and undirected edges representing spatial and functional connections. Use simple and general spatial and functional edge features to build prior graphs, and then use GCN architecture to extract and aggregate features to predict epileptic seizures. While ensuring excellent performance, our method reduces the size of the model so that it can better meet the needs of practical applications of wearable devices. Therefore, it can be used as a standard process to build a generic low-consumption graph network of biomedical signals. In clinical practice, the graph feature we use can serve as an aid in the initial determination of discharge location and type, thus increasing medical interpretability.

## 2. Methodology

### 2.1. Data preprocessing

**Figure 2:**
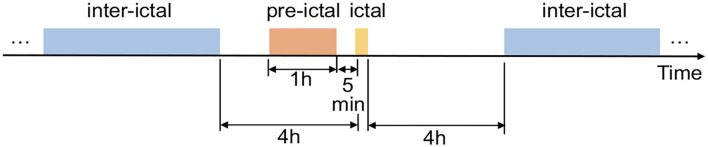
Epileptic brain states.

Currently, The CHB-MIT Scalp EEG Database is one of the few free public datasets of continuous long-term seizures. The dataset marked the onset and end of seizures, and these marks can be used to define the “ictal” for seizure detection. But for the seizure prediction task, we need to define and distinguish between preictal and interictal periods. Due to the small number of subjects, we did not use the EEG from the American Epilepsy Society Seizure Prediction Challenge, but we adopted its rules for dividing pre-ictal and inter-ictal periods because of its relative openness and authority. As shown in Figure 2, 1 h before the seizure is selected as pre-ictal data. It needs to be stressed that pre-ictal data is shifted forward by 5 min as a whole, which allows sufficient predictive time for the patient and avoids the effects of small errors in the onset of a seizure. Moreover, inter-ictal data is limited to 4 h before or after a seizure.

A total of 18 bipolar montages are selected to reduce the effect of noise: Fp1-F7, F7-T7, T7-P7, P7-O1, Fp1-F3, F3-C3, C3-P3, P3- O1, Fp2-F4, F4-C4, C4-P4, P4-O2, Fp2-F8, F8-T8, T8-P8, P8-O2, Fz-Cz, and Cz-Pz. Then, a 1Hz high-pass filter is used to remove low frequency noise, and a 50 Hz and 100 Hz notch filter are used to remove operating frequency noise. Min-Max Normalization is then used to normalize signals to a range of values between [0, 1] or [−1, 1]. Finally, signals are sliced by a fixed length of time (60 s) without overlapping slides.

### 2.2. Feature extraction

Because the graph consists of some nodes and edges that connect these nodes, extracted features can be classified as node features and edge features. The Band Energy, Hjorth parameters, Higher Order Crossings, 1-order difference, 2-order difference, Differential Entropy, and Fractal Dimension are features of nodes, and the geodesic distance and spectral correlation are features of edges.

The Band Energy reflects the level of activity of the cerebral cortex. We calculate the energy of six frequency bands with wavelet packet decomposition. Six frequency bands are Delta (1–4 Hz), Theta (4–7.5 Hz), Alpha (7.5–13 Hz), Lower Beta (13–16 Hz), Higher Beta (16–30 Hz), and Gamma (30–40 Hz).

The Hjorth parameters (Hjorth, 1970) reflect shape features in the time domain, in which activity measures the deviation of the signal amplitude, mobility measures the change in signal slope, and complexity measures the number of standard slopes on an amplitude of signals.


(1) $$\begin{array}{r} Activity=\frac{1}{N}\overset{N}{\underset{n=1}{\sum }}{\left(s\left(n\right)-\mu_{s}\right)}^{2} \end{array}$$

(2) $$\begin{array}{r} Mobility=\sqrt{\frac{var\left(s^{\prime }\left(n\right)\right)}{var\left(s\left(n\right)\right)}} \end{array}$$

(3) $$\begin{array}{r} Complexity=\frac{Mobility\left(s^{\prime }\left(n\right)\right)}{Mobility\left(s\left(n\right)\right)} \end{array}$$

where μ_*s*_ denotes the mean value of signal *s*(*n*), *s*′(*n*) denotes the first derivative of signal *s*(*n*), and var denotes the variance of signal *s*(*n*).

Higher order crossings (HOC) use the number of times the signal passes through the zero point to reflect the degree of signal oscillation (Petrantonakis and Hadjileontiadis, 2009).

1-order difference is the difference between consecutive adjacent two items in the discrete function, which reflects the dynamic relationship between two adjacent frames. 2-order difference is the difference between the 1-order differences of consecutive adjacent two items in the discrete function, which reflects the dynamic relationship between the three adjacent frames.

Differential entropy (DE) is a generalized form of Shannon information entropy on continuous variables.


(4) $$\begin{array}{r} DE=-\int_{a}^{b}p\left(x\right)log\left(p\left(x\right)\right) \end{array}$$

Fractal dimension (FD) can be used to represent the complexity of time domain signals, we have chosen Higuchi Fractal Dimension (Higuchi, 1988), Katz Fractal Dimension (Esteller et al., 2001), and Petrosian fractal dimension (Petrosian, 1995).

Geodesic distances reflect the spatial relationship between scalp electrodes by calculating distances in a spherical coordinate system.


(5) $$\begin{array}{r} Geodesic\;Distance=\cos^{-1}\left(\frac{x_{i}x_{j}+y_{i}y_{j}+z_{i}z_{j}}{r^{2}}\right) \end{array}$$

where *r* is the sphere radius, (*x*_*i*_, *y*_*i*_, *z*_*i*_) and (*x*_*j*_, *y*_*j*_, *z*_*j*_) are two points on the surface of the sphere.

Spectral correlation reflects the energy correlation between scalp electrodes.


(6) $$\begin{array}{r} Spectral\;Coherence=\frac{\left|E\left[S_{ij}\right]\right|}{\left|E\left[S_{ii}\right]||E\left[S_{jj}\right]\right|} \end{array}$$

where *S*_*ij*_ is the cross-spectral density of lead *i* and lead *j*, *S*_*ii*_ and *S*_*jj*_ are the power spectral density of lead *i* and lead *j*.

### 2.3. Graph convolutional networks

Given a graph G=(V,E), where $V=\left\{v_{1},v_{2},\ldots ,v_{n}\right\}$ is a set with *n* nodes and E⊆V×V is a set of edges. Extract the band powers and Hjorth parameters as node features. The adjacent matrix *A*∈ℝ^*n*×*n*^ represents the connection relationship between nodes, which can be used as the edge features. Compute the geodesic distance and spectral correlation to represent the adjacency matrix.

The Graph Fourier Transformation (GFT) is an extension of the discrete Fourier transform to graph, transforming graphs from spatial domain to spectral domain. Signals at all nodes of the graph are ϕ:*V* → ℝ^*n*^ where ϕ_*i*_ is the value at node *v*_*i*_. For a graph ϕ, the GFT and the inverse GFT are computed as:


(7) $$\begin{array}{r} F\left\{\phi \right\}=U^{-1}\phi =U^{T}\phi \end{array}$$

(8) $$\begin{array}{r} F^{-1}\left\{\phi \right\}=U\hat{\phi } \end{array}$$

where *U* is an orthogonalized eigenvector matrix of Laplacian Matrix *L* of an undirected graph *G*.

Convolutions on graphs are an extension of convolution, transforming graphs from spatial domain to spectral domain, and then transforming from spectral-domain back to the spatial domain after performing the convolution operation in the spectral domain. The calculation can be divided into three steps (Ma, 2021):

1. GFT:
  (9) $$\begin{array}{r} F\left\{\phi \right\}=U^{-1}\phi =U^{T}\phi \end{array}$$
2. Convolutions:
  (10) $$\begin{array}{r} g_{\theta }U^{T}\phi \end{array}$$
3. Inverse Graph Fourier Transform (IGFT):
  (11) $$\begin{array}{r} F^{-1}\left\{g_{\theta }U^{T}\phi \right\}=Ug_{\theta }U^{T}\phi \end{array}$$

where θ is the corresponding spectral domain convolution kernel obtained by the spatial domain filter g through the Fourier transform of graphs, and *g*_θ_ is a diagonal matrix whose diagonal elements are θ.

Graph Convolutional Networks (GCN) (Kipf and Welling, 2016) is a simplified network based on ChebyNet (Hammond et al., 2011) proposed by Thomas Kipf. To circumvent the problem of local structure overfitting of ChebyNet on graphs with large node degree distribution, GCN limits the layer-wise convolutional operation to 1:


(12) $$\begin{array}{r} g_{\theta }\left(\Lambda \right)=\overset{1}{\underset{k=0}{\sum }}T_{k}\left(\tilde{\Lambda }\right)\Theta_{k}=\Theta_{0}+\tilde{\Lambda }\Theta_{1}=\Theta_{0}+\left(\frac{2\Lambda_{n}}{\lambda_{max}}-I_{n}\right)\Theta_{1} \end{array}$$

where Λ is the eigenvalue matrix of Laplacian matrix *L*, $T_{k}\left(\tilde{\Lambda }\right)$ is the Chebyshev polynomial of $\tilde{\Lambda }$, and Θ∈ℝ^*c*×*d*^ is a convolution kernel parameters matrix. To further simplify *g*_θ_, approximate maximum eigenvalue λ_*max*_≈2:


(13) $$\begin{array}{r} g_{\theta }\left(\Lambda \right)=\Theta_{0}+\left(\frac{2\Lambda_{n}}{\lambda_{max}}-I_{n}\right)\Theta_{1}\approx \Theta_{0}+\left(\Lambda_{n}-I_{n}\right)\Theta_{1} \end{array}$$

Then, the filtered signal is:


(14) $$\begin{array}{r} Y=g_{\theta }\left(L^{sym}\right)=X\Theta_{0}+\left(-D^{-\frac{1}{2}}AD^{-\frac{1}{2}}\right)U^{T}X\Theta_{1} \end{array}$$

where *L*^*sym*^ is the Symmetric Normalized Laplacian Matrix, *D* is the Degree Matrix of graphs, *A* is the adjacency matrix of graphs, *X*∈ℝ^*n*×*c*^ is the initial attribute matrix of all (*n*) nodes, each node has c-dimensional attributes, and *Y*∈ℝ^*n*×*d*^ is the output of graph convolution. Let Θ = Θ_0_ = −Θ_1_:


(15) $$\begin{array}{r} Y=\left(I+D^{-\frac{1}{2}}AD^{-\frac{1}{2}}\right)X\Theta \end{array}$$

In order to solve the numerical instability, gradient explosion, and dispersion problems that may be caused by multiple iterations, use the renormalization trick: $\tilde{A}=A+I_{n}$, then:


(16) $$\begin{array}{r} Y={\tilde{D}}^{-\frac{1}{2}}A{\tilde{D}}^{-\frac{1}{2}}X\Theta \end{array}$$

Consequently, the layer-wise propagation rule is:


(17) $$\begin{array}{r} f\left(H^{l},A\right)=\sigma \left(\left({\tilde{D}}^{-\frac{1}{2}}A{\tilde{D}}^{-\frac{1}{2}}\right)H^{l}W^{l}\right) \end{array}$$

where *H*^*l*^ is the matrix of activations in the *l*^*th*^ layer, and *W*^*l*^ is a layer-specific weight matrix. σ(·) is an activation function.

## 3. Experiments

### 3.1. Datasets

The CHB-MIT Scalp EEG Database (Shoeb, 2009) is a collection of EEG recordings from 23 pediatric subjects with intractable seizures. We finally select 18 available subjects because of the rare cluster seizures and poor time continuity. Moreover, all signals are sampled at 256 Hz. Files were recorded using the international 10–20 system of EEG electrode positions, most of which have 23 channels.

### 3.2. Architecture for EEG seizure prediction

**Figure 3:**
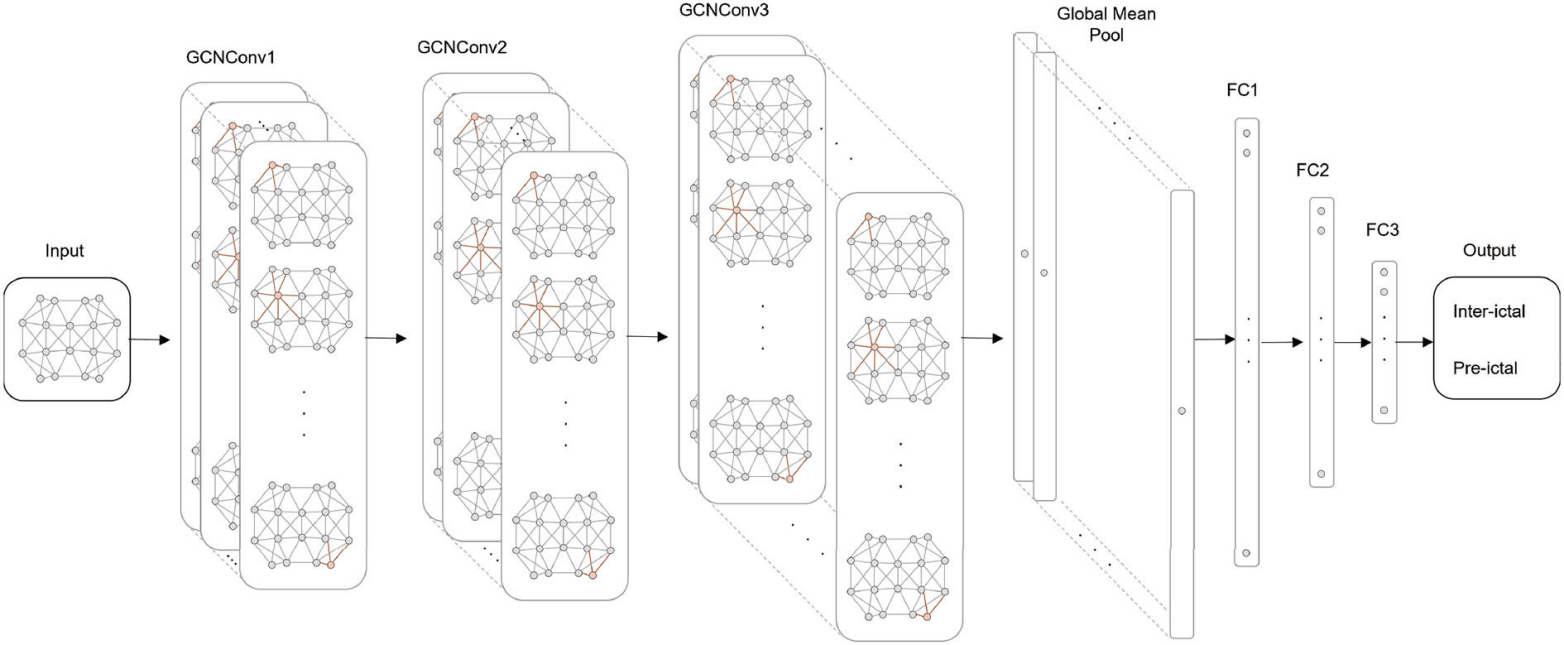
The GCN architecture for seizure prediction.

As shown in Figure 3, we build a GCN architecture for epilepsy prediction, which has seven layers: three GCN convolution layers, one global average pooling layer, and three fully connected (FC) layers. The number of channels in GCNConv is 32, 64, and 128, and the number of neurons in FC layers is 128, 32, and 16. At the top of each GCN convolutional layer, Batch Normalization (BN), and Leaky ReLU activation functions are used to refine node features. Then, the global average pooling layer summarizes the node features of each channel to generate high-level features of the entire graph information. After processing in FC layers, the label probability and class prediction are output through the Sigmoid activation function.

### 3.3. Model training

To obtain more reliable results, we used the Leave One Out Cross Validation (LOOCV) method for each subject. During training, we choose the Adam optimizer whose learning rate is initially set to 0.01 and decreases by 10% after each epoch. We also use the Synthetic Minority Oversampling Technique (SMOTE) (Chawla et al., 2002) and Gradient Harmonizing Mechanism (GHM) (Li et al., 2019a) to ease the numerical imbalance between the two types of samples. We processed each subject's EEG sample by SMOTE and GHM. The ratios of inter-ictal and pre-ictal samples of 18 subjects are as follows: 2:1, 6:1, 17:1, 2:1, 7:1, 15:1, 9:1, 3:1, 6:1, 3:1, 1:1, 2:1, 10:1, 7:1, 2:1, 4:1, 3:1, 1:1, and 6:1. After SMOTE and GHM, all subjects had balanced ratios of 1:1. In addition, we use a 50% probability of randomly discarding neurons in the fully connected layer to avoid overfitting.

### 3.4. Performance metrics

Seizure prediction performance was measured by the mean and SD of sensitivity and AUC on the test set. The sensitivity is defined as follows:


(18) $$\begin{array}{r} Sensitivity=\frac{TP}{TP+FN} \end{array}$$

where the True Positive (TP) and False Negative (FN) can be calculated from the confusion matrix. Furthermore, the Receiver Operating Characteristic Curve (ROC Curve) is obtained by plotting TPR as the horizontal coordinate and FPR as the vertical coordinate at different probability thresholds. The ROC curve considers both sensitivity and specificity and can remain almost constant under different sample distributions. The Area Under ROC Curve (AUC) can be used to quantitatively describe the ROC curve, and classifiers with a higher AUC score perform better.

## 4. Results

### 4.1. Feature selection results

To control the computational cost of the model, we combine the node features in pairs. Table 1 shows the results of the GCN model for feature combinations in the CHB-MIT dataset. Combination Band Energy and Hjorth and Combination Hjorth and FD not only have a sensitivity of over 90% but also have an AUC of over 0.9. Considering the physical significance of sensitivity in seizure prediction, we finally selected the combination Band Energy *and* Hjorth with better sensitivity as the node feature.

**Table 1:** Seizure prediction results of Graph Convolutional Networks (GCN) for feature combinations.

**Feature**	**Sensitivity (**%**)**	**AUC**
**Band Energy**	**89.81 ± 19.13**	**0.88 ± 0.11**
Hjorth	93.90 ± 6.02	0.90 ± 0.11
HOC	88.56 ± 17.97	0.86 ± 0.14
1-order difference	45.90 ± 46.13	0.69 ± 0.18
2-order difference	48.34 ± 46.76	0.67 ± 0.18
DE	58.33 ± 41.69	0.73 ± 0.18
FD	97.70 ± 4.44	0.80 ± 0.18
Band Energy and Hjorth	96.51 ± 4.02	0.92 ± 0.09
Band Energy and HOC	48.05 ± 47.70	0.77 ± 0.22
Band Energy and 1-order difference	84.09 ± 27.44	0.85 ± 0.14
Band Energy and 2-order difference	82.92 ± 27.45	0.85 ± 0.14
Band Energy and DE	88.89 ± 9.84	0.87 ± 0.11
Band Energy and FD	95.27 ± 10.29	0.88 ± 0.12
Hjorth and HOC	94.54 ± 8.26	0.90 ± 0.11
Hjorth and 1-order difference	86.98 ± 25.32	0.88 ± 0.15
Hjorth and 2-order difference	85.78 ± 25.85	0.88 ± 0.16
Hjorth and DE	87.73 ± 22.81	0.89 ± 0.12
Hjorth and FD	96.37 ± 4.88	0.93 ± 0.08
HOC and 1-order difference	79.28 ± 31.52	0.78 ± 0.21
HOC and 2-order difference	79.41 ± 33.20	0.77 ± 0.20
HOC and DE	83.90 ± 28.62	0.76 ± 0.21
HOC and FD	92.50 ± 11.35	0.82 ± 0.17
1-order differenceand 2-order difference	82.54 ± 25.54	0.74 ± 0.16
1-order difference and DE	88.37 ± 12.71	0.68 ± 0.17
1-order difference and FD	78.93 ± 36.23	0.78 ± 0.19
2-order difference and DE	76.47 ± 29.14	0.68 ± 0.17
2-order difference and FD	80.15 ± 33.29	0.77 ± 0.19
DE and FD	73.64 ± 38.54	0.80 ± 0.17

*The meaning of the bold values is “Feature combinations and their performance with not only sensitivity over 90% but also AUC over 0.9”*.

### 4.2. GCN results

Table 2 shows the results of the GCN model for Band Energy *and* Hjorth in the CHB-MIT dataset. The mean sensitivity and AUC for all subjects are 96.51 and 0.92%, and their SD of them are 1.70 and 0.01. The mean sensitivity and AUC of 10 subjects (sub01, sub10, sub11, sub13, sub14, sub16, sub18, sub19, sub20, and sub21) exceed 95%. The mean sensitivity of one subject (sub05) is below 0.9. The mean AUC of six subjects (sub04, sub05, sub06, sub07, sub09, and sub23) are all below 0.9.

**Table 2:** Seizure prediction results of GCN for Band Energy and Hjorth.

**Subject**	**Sensitivity(**%**)**	**AUC**
sub01	97.08 ± 1.18	0.99 ± 0.01
sub02	95.98 ± 1.39	0.90 ± 0.01
sub04	95.56 ± 0.39	0.78 ± 0.02
sub05	83.33 ± 1.18	0.85 ± 0.02
sub06	94.80 ± 6.45	0.82 ± 0.04
sub07	94.65 ± 2.54	0.81 ± 0.01
sub09	98.33 ± 2.04	0.88 ± 0.02
sub10	98.93 ± 1.82	0.98 ± 0.01
sub11	99.17 ± 1.18	0.99 ± 0.00
sub13	99.36 ± 1.11	0.99 ± 0.01
sub14	100.00 ± 0.00	1.00 ± 0.00
sub16	98.89 ± 0.79	0.96 ± 0.01
sub18	99.17 ± 0.83	0.99 ± 0.01
sub19	95.00 ± 4.71	0.95 ± 0.02
sub20	100.00 ± 0.00	0.99 ± 0.00
sub21	98.92 ± 1.08	0.97 ± 0.01
sub22	96.34 ± 2.03	0.93 ± 0.02
sub23	91.59 ± 1.86	0.72 ± 0.03
AVG	96.51 ± 4.02	0.92 ± 0.09

**Figure 4:**
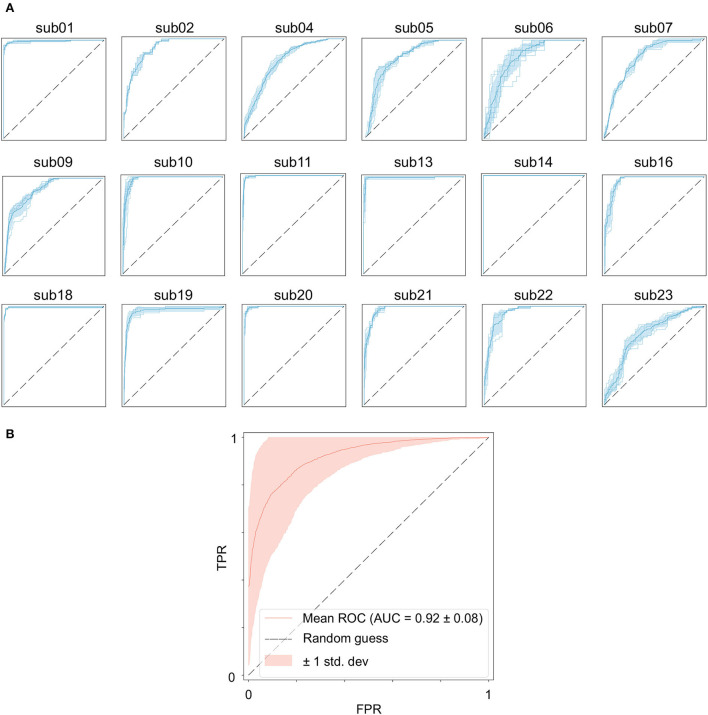
Performance of the GCN model for seizure prediction. **(A)** ROC curves of each subject, **(B)** ROC curves of all subjects.

Figure 4A shows the seizure performance of different patients individually and Figure 4B shows the overall prediction performance of all the 18 patients.

### 4.3. Comparison results

Table 3 shows the comparison between our method and other methods, all of which are evaluated on the CHB-MIT Scalp EEG dataset. It is difficult to directly judge which method is best because of the differences in the patients selected, problem definition, evaluation methods, and performance metrics among the methods. Among all methods, our method uses the longest fixed pre-ictal time (60 min) and a medium number of patients. With these premises, it obtains the second highest sensitivity and the fifth high AUC, which is on par with the performance of similar state-of-the-art methods. Also promising is the achievement of the smallest model size, which can effectively reduce storage space and energy consumption, thus meeting the practical needs of wearable devices better.

**Table 3:** Result of recent studies on predicting seizures on the CHB-MIT dataset.

**Method**	♯ **of cases**	**Evaluation**	**Classifier**	**Sensitivity (%)**	**AUC**	**Model size**	**Pre-ictal duration**
Daoud and Bayoumi (2019)	8	LOOCV	DCAE + Bi-LSTM	99.72	-	27.00	60
Zhang et al. (2019)	23	LOOCV	CNN	92.00	0.9000	33.98	30
Yang et al. (2021b)	13	LOOCV	CNN + ResNet	90.16	0.8909	-	30
Dissanayake et al. (2021a)	23	10-F-CV	CNN	92.45	0.9694	98.66	60
Zhao et al. (2021a)	10	-	CNN + Quan+ Pruning	93.48	0.9770	45.22	30
Dissanayake et al. (2021b)	23	10-F-CV	CNN + LSTM + ChebyNet	95.94	0.9879	289.00	60
Li et al. (2021c)	19	LOOCV	GCN	95.50	0.9380	333.01	15-90
Proposed	18	LOOCV	GCN	96.51	0.9169	15.52	60

## 5. Discussion

### 5.1. Performance degradation

**Figure 5:**
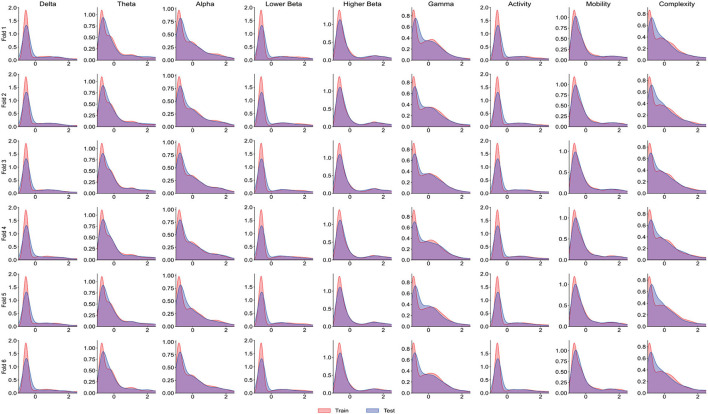
The distribution curve of the training set and test set of sub06.

**Figure 6:**
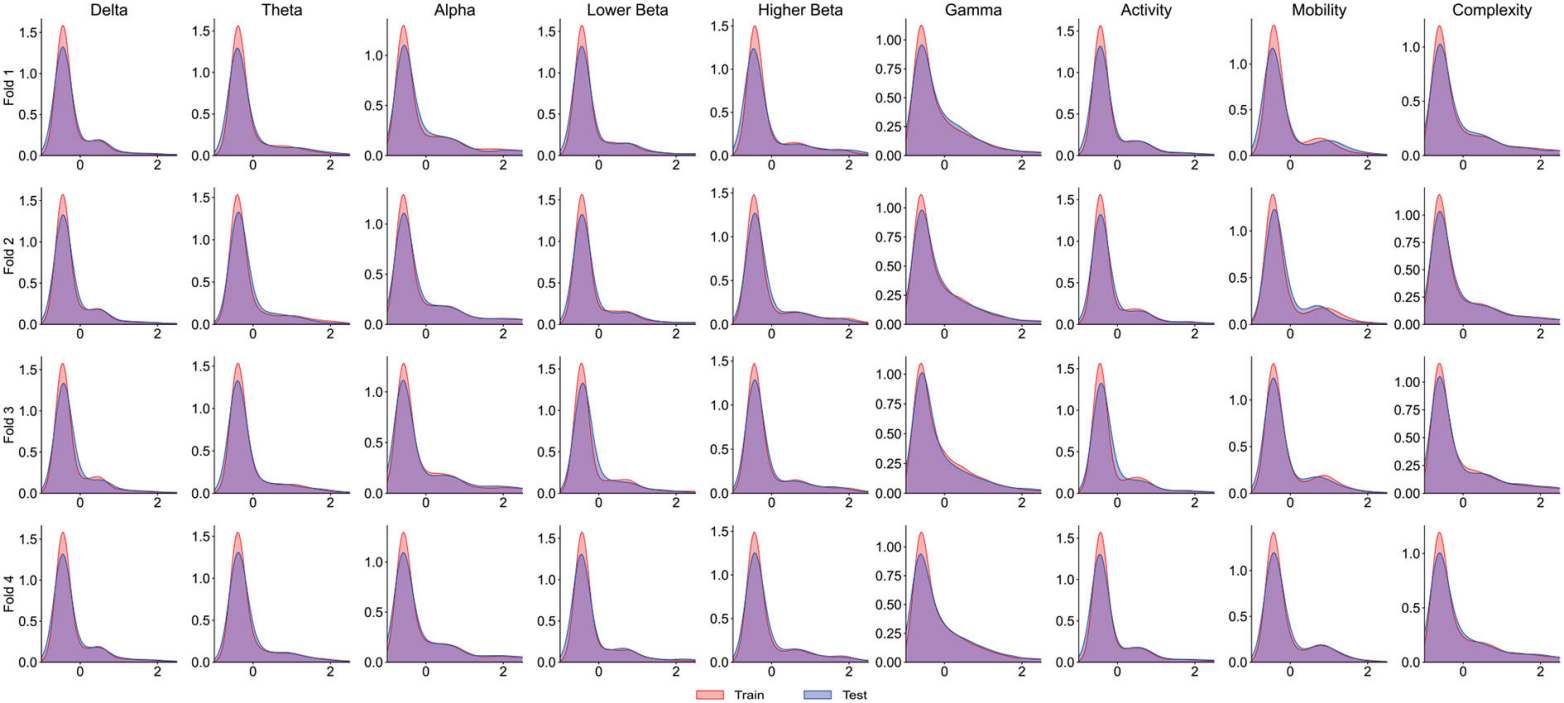
The distribution curve of the training set and test set of sub14.

**Figure 7:**
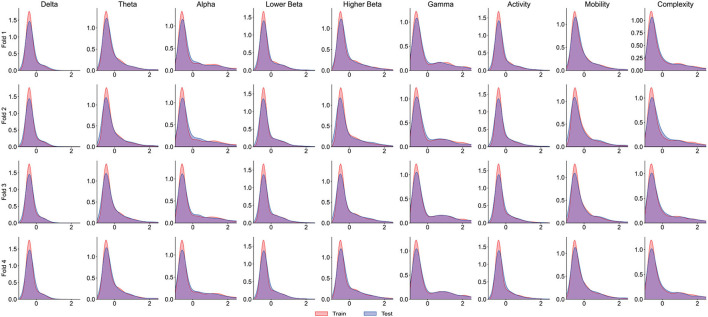
The distribution curve of the training set and test set of sub20.

It is worth noting that the goal of predictive models is to have the ability to apply to new samples, namely generalization ability. The training set is only a partial sample of the sample space. If the sampled training set cannot well reflect the characteristics of the sample space, it is difficult to expect that the model learned from the training set can perform satisfactorily on the unknown test set. In practice, it is generally assumed that all samples are independently and identically distributed. However, EEG is categorized as non-stationary data, and seizure prediction methods suffer from the data drift/covariate drift problem, where the data distribution and characteristics change over time. It can be observed in Figure 4A that there are significant differences in the AUC curves of sub06, and the AUC curves of sub14 and sub20 are completely consistent. Correspondingly, in Table 2, it can be observed that the SD of the prediction results of sub06 is the largest among all patients, and that of sub14 and sub20 is the smallest. By comparing Figures 5–7, it can be found that the distribution of the training set and test set of sub06 is significantly different, and both sub14 and sub20 are identically distributed. This is why the same model architecture has significantly different results between different patients.

Furthermore, within the perspective of raw data, the ratio of sample sizes in the two categories is approximately 17:1, 15:1, and 9:1 for sub04, sub07, and sub09 among 6 subjects with unsatisfactory performance. Besides, most seizures recorded in sub05, sub07, sub09, and sub23 are so concentrated that the inter-ictal data between seizures is missing. In addition, Sub06 recordings have many discontinuities in time.

### 5.2. Performance comparison

Li et al. (2021c) used GCN and LOOCV, and was the closest one in terms of our method. The AUC and longest up-front duration of this method are slightly better than our method, which means that possible subsequent seizures are predicted at an earlier time without increasing too many false alarms. But this also makes it slightly less sensitive than our method, which may miss early warnings for some seizures. Furthermore, to enhance the prediction performance, the method uses a complex deep learning sub-network to synthesize the prior map, which results in a much larger model size than ours, limiting its portable use in everyday life.

### 5.3. Seizure focus

Inter-ictal epileptiform discharges and focal seizures are characterized by a large number of neurons disrupting normal neuronal activity with synchronous discharges, and scalp inter-ictal EEGs are characterized by a large number of neurons collectively superimposed on synchronous oscillations of postsynaptic potentials (Kibler and Durand, 2011). Thus, the electrodes around the epileptogenic zone can record a similar increase in energy, and the corresponding EEG signal is similarly changed at the sites that are reached through the neural network. The strong correlation of functional features in edge features of the graph network can suggest that electrodes correspond to abnormal discharge sites in the patient's inter-ictal period. For temporal lobe epilepsy and frontal lobe epilepsy, which are the most common types of epilepsy, most cases of temporal lobe epilepsy can be roughly located according to EEG interpretation (Blume et al., 2001). In these patients, the strength of correlation can assist clinicians to find the seizure focus, as a supplement to manual reading EEG which mainly depended on the difference in amplitude, frequency, and time sequence of the interictal discharges. Besides, the underlying pathological mechanisms also can be explored by analyzing the characteristics of different subtypes of epilepsy, applying to the seizure origin and the evolution of the typical partial seizures in patients, since the similarity of energy changes was analyzed at the level of total channels not at the level of EEG indicators.

It is important to note that in patients with frontal lobe epilepsy, there are some limitations to scalp EEG. First, inter-ictal discharges in frontal lobe epilepsy are usually widely distributed involving multiple electrodes, and scalp EEG is difficult to detect in patients with deep epileptogenic zones (40% of patients with frontal lobe epilepsy do not record discharges) (Quesney, 1991; Salanova et al., 1993; Bautista et al., 1998), even in patients with medial frontal lobe epilepsy who have focal abnormal discharges at electrodes in the central zone (Blume, 1996).

For example, in the inter-ictal EEG of sub01, based on normal background activity, there are focal paroxysmal abnormalities (spikes and slow waves) issued on both sides of the prefrontal area in the inter-ictal EEG, and intermittent midline rhythms may be common. As shown in Figure 3, there is a strong spectral correlation in the bilateral forehead leads. Additionally, because of the same multifocal issuance in frontal lobe epilepsy, weaker spectral correlations exist in the other leads as well. In the EEG of sub09, unilateral or bilateral synchronous or asynchronous temporal lobe spikes and/or slow waves with conduction to the prefrontal region were observed in the inter-ictal EEG, as well as the existence of discontinuous rhythmic delta wave activity in the occipital region, suggesting that this patient may have temporal as well as occipital lobe epilepsy.

Compared with other CNNs, which can only clinically interpret the amplitude of features, GCN can additionally interpret the connection relationship between features because it retains rich edge features, thus expanding its clinical application. A Chord diagram of functional edge features is introduced in this article to visualize the Spectral correlation. Inspired by the revised Circular Graph (RCG) (Zhao et al., 2019), the visualization draws a circular plot where the montage names are placed on the circumference of the circle. The Spectral correlation values between two montages are drawn as lines connecting the two montages, with their width representing the strength of the energy correlation between two montages.

**Figure 8:**
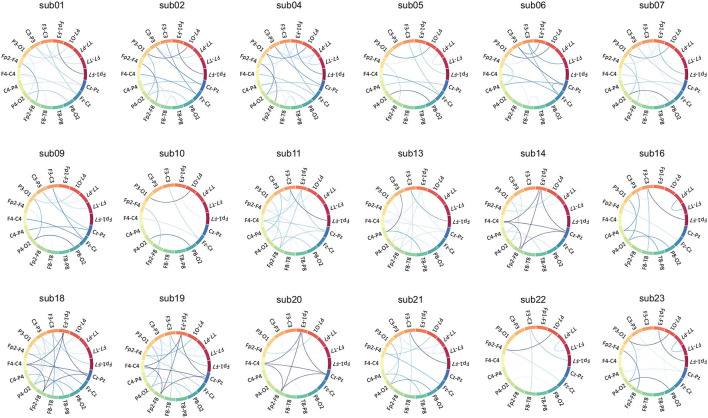
Chord diagram of functional edge features.

The strong spectral correlation results in Figure 8 could indicate similar energy activity in the bilateral occipital leads, which coincides with occipital lobe localization. In contrast, the discharge in the temporal lobe is not shown in Figure 8, probably due to the more restricted discharge in this area and the large difference in energy recorded by the surrounding electrodes.

## 6. Conclusion

The usual conversion of EEG data to a common Euclidean grid structure for processing leads to the loss of adjacent information. To process graph-structured data with richer structural information and to simplify the model size, we propose a new smaller and computationally simple GCN-based architecture for seizure prediction. The proposed method uses only models with a scale of 15.5 k, which matches the state-of-the-art performance of the CHB-MIT scalp EEG dataset. The results show that our method can be regarded as a standard procedure to build a general low-power graph network model for processing similar biomedical signals, which is easier to meet the requirements of EEG-based low-power wearable devices. In addition, graph network functional edge features have different sensitivities for different patients, different discharge locations, and types. This means that in the actual epileptic seizure prediction, we can not only obtain the final prediction result but also make preliminary speculation and judgment on the discharge location and type of the patient to enhance its medical interpretability.

## Data availability statement

Publicly available datasets were analyzed in this study. This data can be found here: PhysioNet, https://physionet.org/, doi: 10.13026/C2K01R.

## Ethics statement

Ethical review and approval was not required for the study on human participants in accordance with the local legislation and institutional requirements. Written informed consent from the patients/participants or patients/participants' legal guardian/next of kin was not required to participate in this study in accordance with the national legislation and the institutional requirements.

## Author contributions

MJ presents the project idea and completes the modeling, experiments, and writing of this manuscript. WL analyses the results from a clinical medical perspective, while QW and ZZ contribute to the design of the study and the editing of this manuscript. JD, LC, and CC contribute to the editing of this manuscript. All authors read and approved the final manuscript.

## Conflict of interest

The authors declare that the research was conducted in the absence of any commercial or financial relationships that could be construed as a potential conflict of interest.

## Publisher's note

All claims expressed in this article are solely those of the authors and do not necessarily represent those of their affiliated organizations, or those of the publisher, the editors and the reviewers. Any product that may be evaluated in this article, or claim that may be made by its manufacturer, is not guaranteed or endorsed by the publisher.
